# Eosinophils in the tumor microenvironment: implications for cancer immunotherapy

**DOI:** 10.1186/s12967-023-04418-7

**Published:** 2023-08-16

**Authors:** Sasan Ghaffari, Nima Rezaei

**Affiliations:** 1 Department of Immunology, H. Lee Moffitt Cancer Center & Research Institute, Tampa, FL USA; 2 Research Center for Immunodeficiencies, Pediatrics Center of Excellence, Children’s Medical Center, Tehran University of Medical Sciences, Tehran, Iran; 3 Department of Immunology, School of Medicine, Tehran University of Medical Sciences, Tehran, Iran; 4 Network of Immunity in Infection, Malignancy and Autoimmunity (NIIMA), Universal Scientific Education and Research Network (USERN), Tehran, Iran

**Keywords:** Eosinophil, T cell, Adoptive cell therapy, Immunotherapy, Tumor microenvironment

## Abstract

Despite being an integral part of the immune response in the tumor microenvironment (TME), few studies have mechanistically elucidated eosinophil functions in cancer outcomes. Eosinophils are a minor population of granulocytes that are mostly explored in asthma and allergic disorders. Their influence on primary and metastatic tumors, however, has recently come to light. Eosinophils’ diverse armamentarium of mediators and receptors allows them to participate in innate and adaptive immunity, such as type 1 and type 2 immunity, and shape TME and tumor outcomes. Based on TME cells and cytokines, activated eosinophils drive other immune cells to ultimately promote or suppress tumor growth. Discovering exactly what conditions determine the pro-tumorigenic or anti-tumorigenic role of eosinophils allows us to take advantage of these signals and devise novel strategies to target cancer cells. Here, we first revisit eosinophil biology and differentiation as recognizing eosinophil mediators is crucial to their function in homeostatic and pathological conditions as well as tumor outcome. The bulk of our paper discusses eosinophil interactions with tumor cells, immune cells—including T cells, plasma cells, natural killer (NK) cells—and gut microbiota. Eosinophil mediators, such as IL-5, IL-33, granulocyte–macrophage colony-stimulating factor (GM-CSF), thymic stromal lymphopoietin (TSLP), and CCL11 also determine eosinophil behavior toward tumor cells. We then examine the implications of these findings for cancer immunotherapy approaches, including immune checkpoint blockade (ICB) therapy using immune checkpoint inhibitors (ICIs) and chimeric antigen receptor (CAR) T cell therapy. Eosinophils synergize with CAR T cells and ICB therapy to augment immunotherapies.

**Supplementary Information:**

The online version contains supplementary material available at 10.1186/s12967-023-04418-7.

## Introduction

Almost 150 years after Paul Ehrlich discovered eosinophils, new regulatory functions are being added to their list [1]. We can categorize the main functions of eosinophils into four groups: (1) effector functions, (2) tissue remodeling, (3) cell interactions (4) immunomodulation [2]. As an innate immune cell, eosinophils exhibit antibacterial [3], antiviral [4] anti-parasitic [5], and anti-tumor [6] effector functions and are involved in inflammatory disorders, allergies and parasitic infections due to T helper type 2 (Th2) function. They are also believed to participate in tissue repair and homeostasis in the steady state and bridge innate and adaptive immunity [7]. In synergy with other chemoattractants, interleukin 5 (IL-5) facilitates eosinophil differentiation in the bone marrow (BM) and guides maturing eosinophils to the peripheral blood. It also activates eosinophils and promotes their tissue survival and degranulation [8]. Circulating eosinophils constitute less than 1% of the total eosinophils. The majority of mature eosinophils reside in peripheral tissues, particularly the mucosal lining of the gastrointestinal (GI) tract. After recognizing endogenous alarmins, pathogen- and damage-associated molecular patterns (PAMPs/DAMPs), or cytokine stimulation, eosinophils activate. Activated eosinophils (CD11b and Siglec-F) [9] experience increased survival and expansion, high adhesion receptor activation and finally release stored mediators [10, 11]. Crystallizable fragment (Fc) ε/γ/α binding to pathogen- or allergen-bound immunoglobulin E/G/A also activates and expands eosinophils during the antigen-specific type 2 immune response. Eosinophils are terminally differentiated cells, but they demonstrate various functions with their pleotropic activities. Despite being traditionally known to participate in type 2 inflammatory responses—such as allergy—these leukocytes take part in type 1 inflammation as well. This polarization depends on environmental cues such as type 1 and type 2 cytokines, which they also secrete [12]. Understanding these interactions is a necessary primary step to studying eosinophils in cancer.

As cancer cells develop, they expand and reshape their surrounding environment. This hypoxic tumor microenvironment (TME), comprised of immune cells, fibroblasts, endothelial cells, cancer cells, etc. is a specialized domain with intricate interactions between the resident or recruited cells and cancer cells. The TME contributes to cancer progression and metastasis by providing a nest conducive to tumor cell grow [13]. Every innate and adaptive immune cell infiltrates TME in order to suppress tumor growth, which results in different outcomes. The cross-talk of tumor cells with resident cells is crucial to patient outcomes, which makes investigating such interactions with every resident cell critical. The role of eosinophils in tumor progression is being explored. First described in the 19th century, tumor-associated tissue eosinophilia (TATE), in which eosinophils infiltrate tumor sites, is a recurring theme in cancer patients [14]. In solid tumors, TATE predicts favorable prognosis and is inversely correlated with tumor metastasis and stage [15]. The precise function of eosinophils in killing or promoting cancer cells is debated, but recent studies are mostly in favor of their anti-tumorigenic role. These results are also applicable in other eosinophil-related disorders such as allergic asthma where eosinophil depletion as a viable strategy could have negative long-term impact on tumorigenesis in the patients. Combining eosinophil heterogeneity of function with their ability to cross-talk with TME cells and their prognostic value justifies the need to investigate the role of eosinophils in cancer. Because these findings provide the necessary knowledge for overcoming the current challenges and drawback in cancer immunotherapy, we summarized key studies pertaining to the function of eosinophils in cancer outcome.

## The development and biology of eosinophils

Eosinophils are known as granulocytic leukocytes, differentiated from myeloid progenitors in the BM [5, 16]. Eosinophil differentiation is controlled by four main cytokines, including IL-3, granulocyte–macrophage colony-stimulating factor (GM-CSF), stem cell factor (SCF), and most importantly IL-5 [5]. The early effect of C/EBPα, C/EBPε, GATA-1 and -2, IRF8, and PU.1 transcription factors is even more pivotal for eosinophilic lineage commitment [17]. After a short 3 to 18-hour period in the peripheral blood, mature eosinophils migrate to tissues such as the GI tract, adipose tissue, lungs, and thymus with the aid of their numerous receptors. In baseline, non-pathological conditions, eosinophils are primarily drawn by CCL11 (eotaxin-1) to the GI tract and then other tissues in fewer numbers. In response to inflammation, they are recruited to inflamed tissues under the chemoattractive influence of Th2 cytokines (IL-4, IL-5, IL-13), chemokines such as eotaxins, 5-oxo 6, 8, 11, 14-eicosatetraenoic acid (5-oxo-ETE) and prostaglandin-D2 (PGD2), and finally IL-33 [18]. CCL11, which is the most potent eosinophil chemotactic factor, is produced by epithelial cells and fibroblasts and, to a lesser extent, by other leukocytes. Eosinophils consist of different subsets. Resident eosinophils (CD125^int^Siglec-F^int^CD62L^+^CD101^low^) that are drawn to tissues in the steady state and possess regulatory functions and recruited inflammatory eosinophils (CD125^int^Siglec-F^hi^CD62L^–^CD101^hi^) that promote inflammatory responses [19]. Eosinophils infiltrate a tissue using extracellular matrix-degrading matrix metalloproteinases (MMPs) that not only help with the infiltration of other immune cells but also reshape the connective tissue [20].

Cell-to-cell contact or indirect mediation by their mediators allow eosinophils to influence other resident cells in a tissue [21]. The stored mediators of eosinophils and their intra and extracellular receptors possess key roles in eosinophilic functions and interaction with other cells (cross-talk). Eosinophil mediators are a plethora of preformed cytokines, chemokines, cationic proteins, growth factors, neuro-mediators, and lipids stored in eosinophil granules (Table 1). Mediator storage allows for rapid eosinophil response without the need for de novo protein synthesis, which makes them unique compared to other immune cells [2]. Eosinophil secretory organelles are small primary granules (also immature specific granules), large specific (secondary) granules, and lipid bodies that house a variety of cytokines, chemokines, receptors, growth factors, and lipids with distinct functions (Table 1). Such a collection of mediators testifies to the diverse role of eosinophils. They owe their main morphological feature to specific granules that contain highly basic cationic proteins such as major/main basic protein (MBP) in their electron-dense crystalloid core and eosinophil peroxidase (EPX/EPO), eosinophil cationic protein (ECP), and eosinophil-derived neurotoxin (EDN) in their electron-lucent matrix whose acidophilic nature stains red with acid dyes such as eosin [5]. Charcot–leyden crystal (CLC) proteins (galactin-10), which interact with eosinophil lysophospholipases and cause inflammation and Th2 stimulation, are stored in primary granules but mostly in the peripheral cytoplasm [22]. The prominent presence of large secondary granules helps microscopically distinguish eosinophils from other granulocytes. Eosinophils express a range of receptors needed for activation, proliferation, trafficking, adhesion, pattern recognition, and degranulation as well. These allow them to migrate to tissues and interact with resident cells and molecules making them a player in the intricate cell cross-talk in tissues [23].

**Table 1:** Important eosinophil receptors and granules and their respective functions

	Name			Components	Function
Granule	Primary			CLC	Causes inflammation and stimulates Th2 cells, has lysophospholipase activity
	Secondary (specific, crystalloid, crystalline, alpha)		Crystalline core	MBP	Neutralizes heparin, is bactericidal by changing the negative charge of lipid bilayer and disorganizing it, toxic for helminths and airway epithelium, activates complement system, stimulates basophil/mast cell for histamine secretion
				GM-CSF	Eosinopoiesis induction
				IL-4	Th2 and M2 polarization, IgE class switching, CCL11 secretion
				Cathepsin D, hydrolase, glucuronidase	ECM degradation
			Matrix	ECP ribonuclease	• Forms toxic pores on target cell membranes and is bactericidal, antiviral, anti-helminth • Sends signals to other cells, degranulates mast cells
				EPX (EPO)	Cytotoxic, generates reactive oxygen species (ROS) for oxidative damage
				EDN ribonuclease	Possesses antiviral activity
				IL-2	Crucial for the expansion of all T cells
				IL-6 and APRIL	Support long-lived plasma cells and B cell development
				IL-13	Helps IL-4 with M2 polarization and IgE class switching
				IL-10	M2 polarization, immune cells’ suppression
				IL-5, SCF	Eosinopoiesis, eosinophil activation, recruitment
				IFN-γ	Th1 and M1 differentiation, CD8 + T cell stimulation, vessel normalization, antiviral, antibacterial, IL-12 production, tumor suppression
				IL-12	Th1 differentiation, IFN-γ production by NK cells and Th1 cells, Th2 inhibition
				TNF, IL-1β	Have strong proinflammatory activities
				TGF-α	Promotes epithelial wound healing and cell proliferation
				TGF-β	• Plus IL-4: Th9 differentiation • Plus IL-2: Treg differentiation • plus IL-6/IL-21: Th17 differentiation Exacerbates tissue fibrosis IgA class swithcing
				Histaminase	Degrades histamine
				Collagenase, elastase, catalase, esterase	ECM degradation
				NGF, PDGF, VEGF, EGF	Growth factors for proliferation of various cells
				CCL7 (MCP-3)	A potent chemoattractant for monocytes, eosinophils, basophils, DCs, NK and T cells
				CXCL1 (Groα)	Neutrophil chemoattractant, involved in angiogenesis and tumorigenesis
				CCL11 (eotaxin-1)	The most important eosinophil chemoattractant, induces piecemeal degranulation (PMD)
				CCL13 (MCP-4)	A major eosinophil, basophil, monocyte, T cell chemoattractant
				CCL3 (MIP-1α)	Attracts macrophages, lymphocytes, and eosinophils
				CCL5 (RANTES), CXCL9, CXCL10 (IP-10), CXCL16	Secreted from activated eosinophil to recruit Th1, CD8 + T, and NK cells
				CXCL12 (SDF-1)	A weak eosinophil chemoattractant
				CCL17, CCL22	Th2 recruitment to tissue, Treg recruitment (CCL22)
				CCL20 (MIP-3α)	Induces eosinophil migration and activation
				CCL9 (MIP-1γ)	Recruits DCs and macrophages to Peyer’s patches
	Lipid bodies			LTC4, LTD4, LTE4	Induce bronchoconstriction, mucus production, airway inflammation, eosinophil lineage commitment
				PGE1, PGE2	Smooth muscle relaxant and vasodilator
				TXB2	Vasoconstriction and platelet aggregation
				15-HETE	Vasoconstriction and angiogenesis promotion
				PAF	Eosinophil chemoattractant, increases LTC4 production and eosinophil adhesion
				COX, 5-lipoxygenase, LTC4 synthase	Esterify arachidonic acids and produce eicosanoids such as PGs and LTs
Receptors	Cytokine and growth factor receptors			IL-2Rα/β (CD25/CD122)	Binds to IL-2 that activates eosinophils to release EPX and induces chemotaxis
				IL-3Rα/β (CD123/CD131)	Binds to IL-3 that helps with eosinopoiesis
				IL-4Rα/β (CD124/CD132)	Binds to IL-4 that activates eosinophils
				IL-5Rα/β (CD125/CD131)	• IL-5Rα binds to IL-5 • IL-5Rβ transfers the signal
				IL-9Rα/β (CD129/CD132)	Binds to IL-9 that promotes eosinophil, mast cell, Treg, Th17 growth
				IL-13Rα1 (CD213a1)	Binds to IL-13 that promotes P-selectin expression on eosinophils
				IFN-γR1 (CDw119)	Binds to IFN-γ which boosts eosinophil anti-tumor activity
				TGF-βR1	Binds to TGF-β
				TNFαR1 and 2 (CD120a, b)	Bind to TNF-α that delays eosinophil apoptosis
				IL-10R^b^	Binds to IL-10 cytokine family such as IL-22 (important in antimicrobial peptide production) and IFN-λ (antiviral)
				IL-13Rα1 (CD213a1)	Binds to IL-13
				IL-17 A/F R (IL-17RA/IL-17RC)	Bind to IL-17 A and IL-17F of Th17 to cause IL-1β and IL-6 production
				IL-18R	Binds to IL-18 that increases LFA-1 and ICAM-1 expression
				IL-23R (IL-23R/IL-12Rβ1)	Binds to IL-23 that increases IL-17RA expression and recruits eosinophils to lungs
				IL-27R (gp130/WSX-1)	Binds to IL-27 that prolongs eosinophil survival by reducing apoptosis
				IL-31R (IL-31RA/OSMRβ)	Binds to IL-31 that activates eosinophils
				TSLPR	Binds to TSLP cytokine that modulates eosinophil survival and promotes eosinophilia
				GM-CSFRα/β (CD116/CD131)	Binds to GM-CSF to help with eosinophil lineage commitment and survival
				SCFR (KIT) (CD117)	Binds to SCF to help with eosinophil lineage commitment and survival
				IL-25R and IL-33R (ST2)	Bind to IL-25 and IL-33 that modulate eosinophil survival and promote eosinophilia
	Fc receptors			FcεRI	High-affinity IgE receptor that does not activate eosinophils
				FcεRII (CD23)	Low-affinity receptor for IgE
				FcγRII (CD32)	Binds to IgG and triggers eosinophil activation
				FcαRI (CD89)	Binds to IgA and triggers eosinophil activation and prevents apoptosis
				FcγRI (CD64), FcγRIII (CD16)	Not constitutively expressed, upregulated by IFN-γ, C5a, PAF
	Adhesion receptors	Integrin		CD11/CD18 (LFA1)	Binds to ICAM-1 (CD54) and -2 (CD102) for infiltration in airways
				CD11b/CD18 (CR3)	Binds to ICAM-1 and fibrinogen, binds to β−glucan to induce EDN release against fungi
				CD11c/CD18 (CR4)	Binds to iC3b
				CD49d/CD29 (VLA4)	Binds to VCAM-1 to carry out extravasation
		Selectin		L-selectin (CD62L)	Binds to Sialyl Lewis X of GlyCAM-1, CD34, MadCAM-1 on endothelium for eosinophil tethering before integrin activation
		Others		PSGL-1	Its Sialyl Lewis X (CD15s) binds to P-selectin (CD62P) and E-selectin (CD62E) on endothelium for eosinophil tethering before integrin activation
	Inhibitory receptors			Siglec-8	Binds to sialic acid and induces eosinophil death
	Secretory proteins			VAMP-2, -7, -8, STX-17	Located in granules and Sombrero vesicles, form SNARE complexes with STX-4 and SNAP-23, crucial for exocytosis and PMD
				Syntaxin-4 (STX-4), SNAP-23	Located in plasma membrane, form SNARE complexes with VAMP-2, -7, -8, and STX-17, crucial for exocytosis and PMD
	Pattern recognition receptors (PRRs)			TLR-1, -2, -4, -5, -6	Present on cell surface and detect bacterial proteins, lipoproteins, and polysaccharides
				TLR-3, -7, -8, -9, -10	Present in endosome and detect viral nucleic acids
				CLRs	Detect fungal components
				RLRs	Present in cytosol to detect viral dsRNA
				RAGE	Binds to HMGB1 and promotes eosinophilia
				NLRs	Present in cytosol and detect bacterial peptidoglycan
	Chemokine receptors			CCR1 (CD191)	Binds to CCL3 (MIP1α), CCL5 (RANTES), CCL7 (MCP3), PAF
				CCR2 (CD192)	Binds to CCL7, CCL8 (MCP2)
				CCR3 (CD193)	• Binds to eotaxins (CCL11, CCL24, CCL26) that greatly enhance eosinophil chemoattraction with IL-5 • Binds to CCL5 (RANTES), CCL13 (MCP4), CCL7, CCL8
				CCR4 (CD194)	Binds to CCL17 and CCL22
				CCR5 (CD195)	Binds to CCL1, CCL5, CCL7
				CCR6 (CD196)	Binds to CCL20
				CCR8 (CD198)	Binds to CCL1 that is bactericidal and recruits eosinophils
				CCR9 (CD199)	Binds to CCL25
				CXCR2 (CD182) (IL-8Rβ)	Binds to CXCL1
				CXCR3 (CD183)	Binds to CXCL9, CXCL10
				CXCR4 (CD184)	Binds to CXCL12
				FPR1	Binds to microbial peptides that induces leukocyte chemotaxis
	Complement receptors			CR1 (CD35)	Binds to C3b, C4b, iC3b, C1q to activate eosinophils
				CR3 (CD11b/CD18)	Binds to iC3b and ICAM-1
				C3aR, C5aR, CR4, CD103	Bind to complement components that activate and attract eosinophils
	Eicosanoid receptors			CysLT1R, CysLT2R, LTB4R, PGD2R, PGE2R, PAFR, fMLPR	Bind to lipid mediators such as LTs and PGs
	MHC-II			Plus CD80/86 or CD40	MHC-II presents T cells with antigen peptides and CD80/86 or CD40 provide co-stimulation
				HLA-DR	Upregulated in activated eosinophils

*ECP* eosinophil cationic protein, *EPX* eosinophil peroxidase, *EDN* eosinophil-derived neurotoxin, *15-HETE* 15-hydroxyeicosatetraenoic acid, *LTC/D/E* leukotriene C4/D4/E4, *PAF* platelet-activating factor, *PGD2/E2* prostaglandin D2/E2, *TSLP* thymic stromal lymphopoietin, *CLRs* C-type lectin receptors, *RAGE* receptor for advanced glycation end-products, *NLRs* NOD-like Receptor, *RLRs* RIG-I Receptor, *RANTES* regulated on activation, normal T cell expressed and secreted, *Siglec-8* sialic acid-binding Ig-like lectins 8, *MHC-II* major histocompatibility complex-II

## Eosinophil functions in non-pathological and pathological conditions

Although eosinophils are primarily known for their role in allergic disorders and anti-parasitic function, especially against helminths, they are involved in many non-pathological activities as well [24]. In the steady state, resident eosinophils perform functions related to tissue development/maintenance, tissue regeneration and remodeling [25, 26], metabolism, and immune homeostasis [18], all of which are integral to innate immunity. Contrary to cytotoxic mediators, eosinophil cytokines and growth factors improve tissue health when produced in moderation. The GI tract benefits the most from these non-pathological functions that include plasma cells (PCs) maintenance, IgA class switching and mucus production [27], regulatory T cell (Treg) and T helper 17 (Th17) differentiation and regulation, Peyer’s patch development, and alteration of gut microbiota composition [28]. A number of other functions in various organs are performed by eosinophils: wound healing, epithelial remodeling and immune homeostasis in the respiratory system [29], maintenance of alternatively activated (M2) macrophages that contribute to insulin sensitivity, glucose tolerance, and inflammation regulation in the adipose tissue [30] and maintenance of PCs via IL-6 and APRIL in the bone marrow. Apart from increasing PC survival and IgA production, eosinophils serve in humoral immunity through the priming of B cells and the early generation of antigen-specific IgM as well [31]. By discarding apoptotic cells and participating in the elimination of self-reactive T cells—negative selection—via indoleamine 2,3-deoxygenase (IDO) secretion, eosinophils serve to maintain thymus homeostasis [32].

Eosinophils carry out their duty in pathological situations by secreting their mediators, stimulating other cells, using antibody- and complement-mediated cytotoxicity, performing phagocytosis, or utilizing their receptors and remodeling tissues [2]. Pattern-recognition receptors (PRRs) are crucial in activating degranulating eosinophils in pathogen-infested sites to allow eosinophils to participate in innate immunity. The main cytotoxic contents of an eosinophil are MBP and eosinophil-associated RNases such as ECP and EDN that induce cell necrosis and tissue inflammation in both invading and host cells. The secondary granules release proteins that exert direct cytotoxic effects on bacteria and viruses [33]. Eosinophils chiefly employ piecemeal degranulation (PMD) as their most common degranulation strategy. PMD, first described in basophils and then mast cells, allows granule contents to be transferred to the cell surface via vesicular transport in a controlled manner. This is while the cells stay viable and alert. The secretory eosinophil sombrero vesicles (EoSVs), which are not granules, secrete these mediators. If the strength and duration of the stimulation are above a certain threshold, eosinophils die and release both intact granules and free mediators via cytolysis. Ejected whole granules are capable of remaining viable in tissue for weeks and release their contents upon stimulation [34]. Exocytosis of mediators is another less frequent form of direct degranulation. Finally, eosinophil extracellular traps (EETs), which are amalgamations of cationic proteins and mitochondrial DNA that capture and kill pathogens, are another type of mediator release [35].

**Fig. 1:**
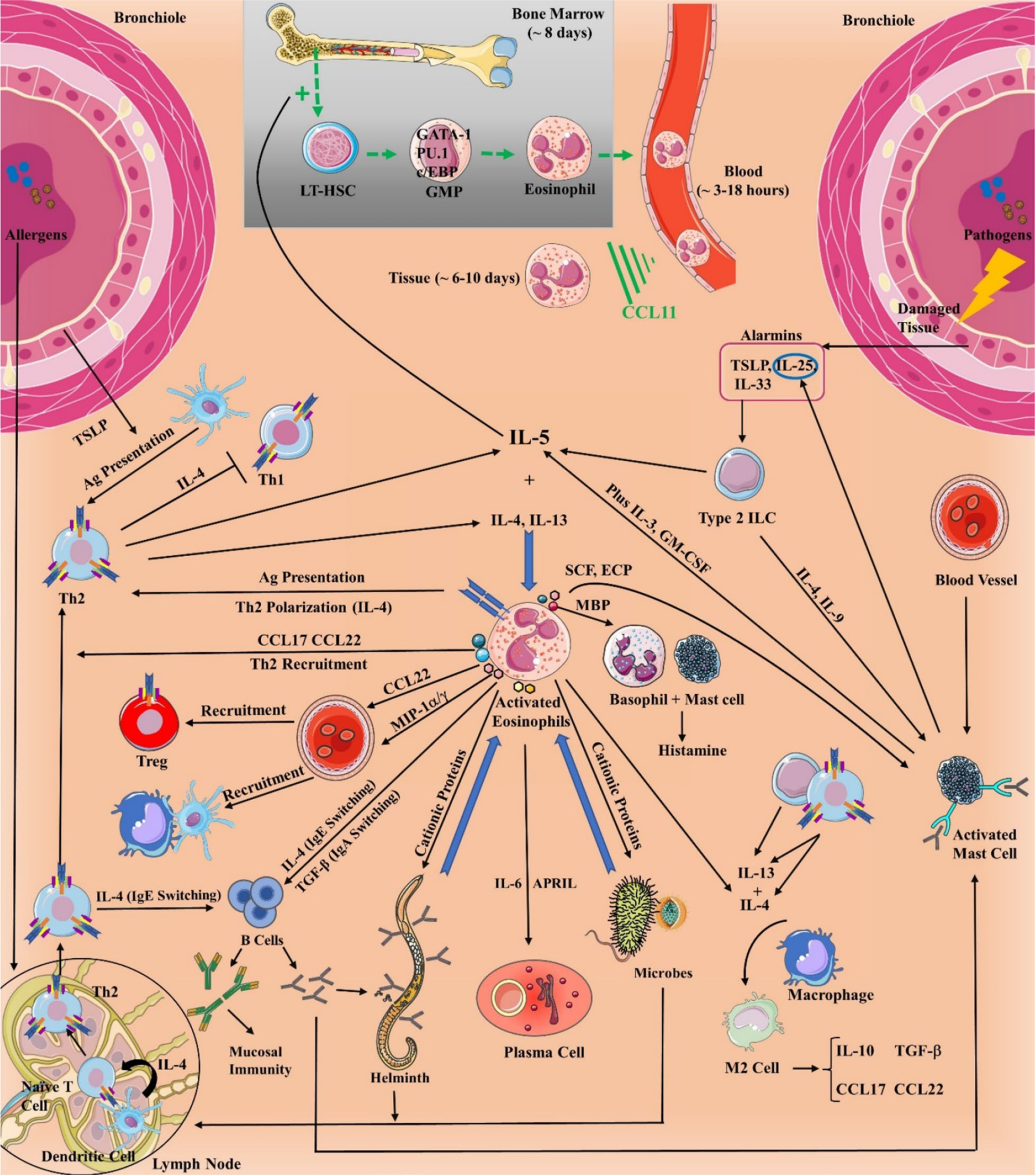
Eosinopoiesis, its transcription factors, and eosinophil chemotaxis to the blood and tissue and their function in type 2 immunity. IL-5 is pivotal in stimulating eosinopoiesis and recruiting mature eosinophils to tissue with CCL11, which is produced from fibroblasts and epithelial cells. Type 2 innate lymphoid cells (ILCs) and dendritic cell- (DC) activated Th2 cells are important eosinophil activation and recruitment routes while pathogens directly stimulate eosinophils. Damaged endothelial and epithelial cells, fibroblasts, and adipocytes kickstart the process by secreting alarmins that stimulate IL-5 production from type 2 ILCs. On the other hand, mature DCs help with T helper 2 (Th2) polarization. Eosinophils typically release their mediators via the classical exocytosis, or the release of intact granules upon their lysis (cytolysis), or piecemeal degranulation (PMD) which is the most popular method. *LT-HSC* long-term hematopoietic stem cell, *GMP* granulocyte-monocyte, *MBP* major basic protein, *SCF* stem cell factor, *ECP* eosinophil cationic protein

Eosinophilic interactions with innate and adaptive immune cells and their receptors enhance and regulate immune and tissue responses, particularly in type 2 immunity [21]. After extravasating into the tissue, eosinophils release chemoattractants to recruit Th2 cells (Fig. 1). Although not a professional antigen-presenting cell, antigen-exposed eosinophils stimulate primed T cells via major histocompatibility complex-II (MHC-II) and CD80/CD86 costimulatory molecules, denoting their role in adaptive immunity. Activated eosinophils polarize naïve CD4 + T cells to Th2 cells, causing a positive feedback loop to eosinopoiesis and Th1 suppression [36]. They also provide Th2 chemotaxis by secreting CCL17 and CCL22. Th2 and type 2 innate lymphoid cells (ILCs) produce cytokines (e.g. IL-5) that are crucial in eosinophil expansion, activation, survival, and trafficking [37, 38]. Alarmins such as thymic stromal lymphopoietin (TSLP), IL-25 (IL-17E), and IL-33 stimulate type 2 ILC secretion of IL-5 to create eosinophilia [21]. IL-5 is primarily produced by Th2 and ILC2 cells and, in smaller quantities by mast cells, natural killer (NK) and NKT cells, eosinophils, etc. Both eosinophils and mast cells recruit each other in order to improve their function and survival. MBP and tryptase are two mediators released by eosinophils and mast cells, respectively. MBP and ECP stimulate histamine secretion by mast cell and basophil, while SCF heavily regulates mast cell differentiation, growth, and recruitment [39, 40]. Moreover, eosinophils have multifunctional effects on B cells such as survival, proliferation, immunoglobulin secretion, lifespan extension of naïve B cells, and immunoglobulin isotype switching, which is direct or indirect via Th2 cells [41]. Eosinophils maintain B cell development and PC survival through IL-6 and APRIL [42]. In view of eosinophils’ pivotal role in orchestrating the immune system, they could serve as a potential target for immunotherapy.

## Eosinophils in the tumor microenvironment

The consequences of eosinophil participation in TME are still elusive. Numerous studies have investigated the association between TATE and patient outcomes (Additional file 1). TATE is generally associated with a favorable prognosis for solid tumors [15]. The direct effect of eosinophil on cancer cells, however, has received less attention. Despite the fact that the presence of eosinophils in tumor sites is neglected in most literature, eosinophilia in the blood and/or the TME is an inseparable part of many cancers. Eosinophils are sometimes drawn to tumor cells independent of their usual chemokines. Although multiple studies across different neoplasms have shown elevated levels of eotaxins correlate with TATE [43–45], unlike homeostatic or allergic conditions, eosinophils can migrate to tumor sites without requiring CCR3 [9]. Early recruitment of eosinophils to solid tumors can be independent of CD4 + T or ILC2 cells as well. IL-5–producing malignant cells can recruit circulating eosinophils and increase eosinopoiesis [46]. Eosinophils are also capable of responding to the chemotactic factors of necrotic tumor cells [47].

**Fig. 2:**
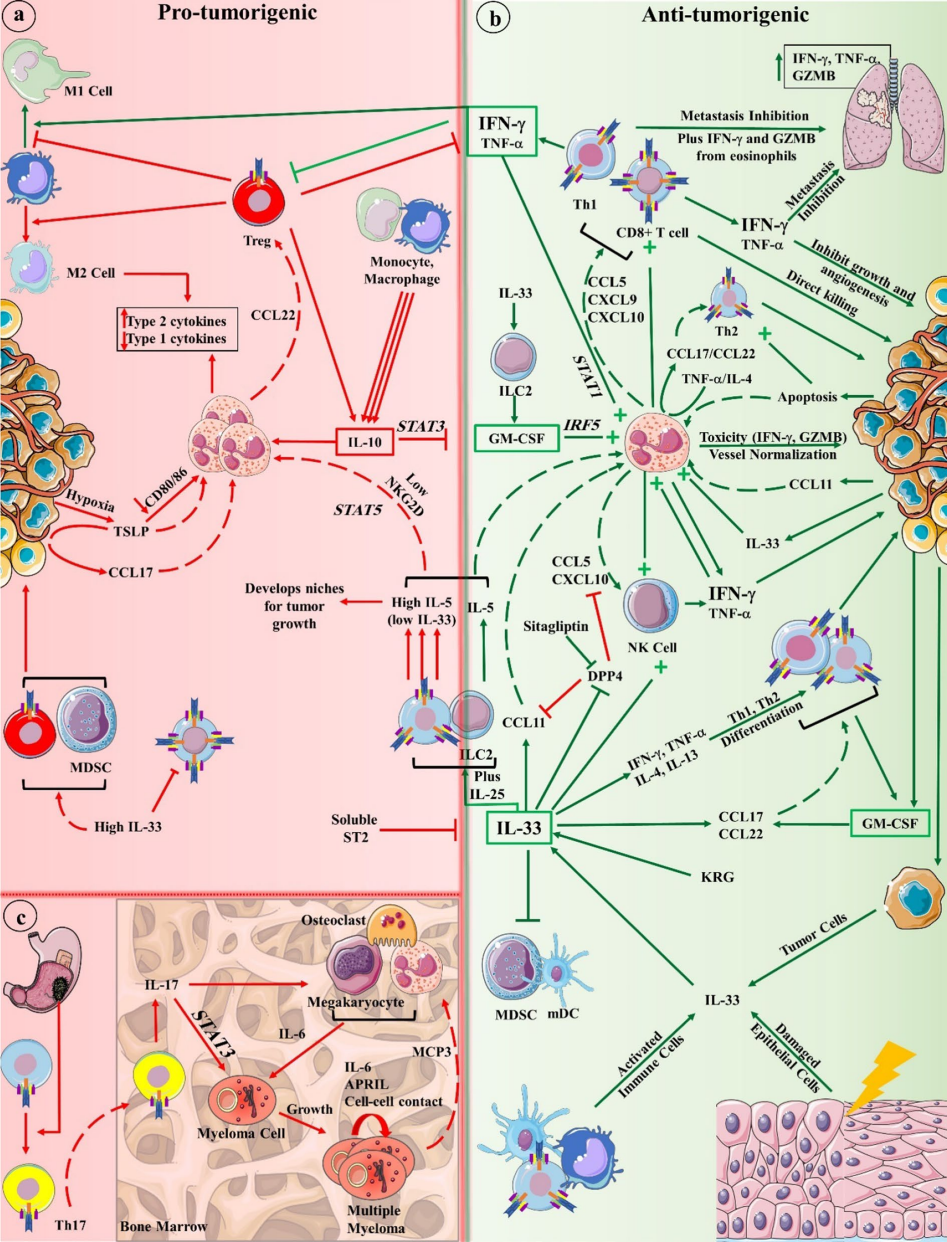
Eosinophil interactions in the tumor microenvironment could be pro-tumorigenic (**a**) or anti-tumorigenic (**b**). IFN-γ and TNF-α (to a lesser extent) activate eosinophil tumor suppression mechanism through STAT1 pathway and activate other immune cells, such as CD8 + T cells, Th2 cells, and NK cells to attack primary or metastatic tumors. GM-CSF also positively activates eosinophils by IRF5 transcription factor. IL-33, produced by tumor, immune, or damaged cells, exhibits a plethora of functions that result in tumor regression. In the absence of IFN-γ, eosinophil-recruited Tregs are central to further suppress type 1 immunity and provide a niche suitable for tumor cells’ growth. In multiple myeloma, eosinophils assist myeloma cell growth, especially in earlier stages, by providing IL-6 and APRIL with other cells such as the stromal cells of the bone marrow or osteoclasts (**c**). Some gut microbiota can exacerbate this situation by stimulating naïve T cell differentiation to Th17 cells that secrete IL-17. Green and red lines point tumor suppression and progression, respectively. Dashed lines indicate cell recruitment. *KRG* Korean red ginseng, *MCP3* monocyte chemotactic protein-3, *GZMB* granzyme b

The interplay between the adaptive immune response and eosinophils is central to decrypting their effect on cancer. Adaptive immunity is balanced between two crucial types of pro- and anti-inflammatory immunity to restore and maintain homeostasis: type 1 and type 2 immunity, with each one being capable of suppressing the other. Type 1 and 2 immunity are centered around Th1 and Th2 cells that are differentiated from naïve CD4 + T cells in the presence of IFN-γ/IL-12 or IL-4/IL-10/IL-13, respectively. By initiating CD8 + cytotoxic T cell- (CTL) mediated immunity, Th1 cells possess stronger Ag-specific cytotoxicity against tumor cells than Th2 cells that tend to induce tumor necrosis. Although type 2 immunity is typically associated with allergic inflammation or parasitic infections, it can also eradicate tumors [48, 49]. Eosinophil commitment to Th1 response was previously shown as well where these leukocytes produced CXCL9, CXCL10, CXCL11, and CCL5 under the effect of TNF-α and IFN-γ, which recruit T and NK cells to eradicate tumor [50]. TNF-α, and more importantly IFN-γ, can inhibit tumor growth, metastasis, and angiogenesis, induce M1 macrophage polarization and Treg fragility as well as tumor senescence [51]. Th2 cells can also hinder tumor progression by enhancing eosinophils [52]. In CTL-resistant melanoma, Th2 cells can clear lung metastases via eosinophil tumor infiltration and degranulation in an eotaxin- and STAT6-dependent manner [53]. Depending on microenvironment cytokines, eosinophils can promote either a Th1 or Th2 response. In the presence of TNF-α/IL-4, eosinophils produce CCL17 and CCL22 Th2 chemokines while, when stimulated with TNF-α/IFN-γ, they secrete CXCL9 and CXCL10 Th1 chemokines [54]. The latter occurs in a STAT1–mediated pathway, which is known to inhibit tumor occurrence [54, 55]. As with the established macrophage polarization, activated eosinophils are polarized in the presence of Th1 or Th2 cytokines. A recent study showed the plasticity of eosinophils where eosinophils experienced transcriptional changes toward anti-inflammatory functions such as wound healing and cell migration upon interacting with apoptotic cells. Apoptotic cells suppress IFN-γ-mediated eosinophil activation and polarize them toward IL-4-induced type 2 responses including tissue repair and remodeling [56]. The following outlines discuss how eosinophils impact TME components (Fig. 2).

### Tumor cells

Two major routes by which eosinophils interact (in)directly with tumor and TME cells exist: (1) the release of cytokines and granule contents that influence TME and tumor cells, (2) the binding of eosinophil receptors to TME and tumor cells, which alters cancer outcome. Hence, preclinical studies focused on how eosinophils and their relevant mediators act on tumors and the resident TME cells. We categorized the following sections based on the cells that eosinophils interact with either directly or through their prominent mediators. In addition to regulating immune types, eosinophils exhibit direct, tumoricidal activities with the secretion of reactive oxygen species (ROS)—such as superoxide and EPX—MBP, ECP, TNF-α, granzyme B, and granzyme A [52, 57–60]. TNF-α, for instance, is able to kill tumor cells in a dose-dependent manner but only if TNFR1 is expressed on the target cells [61].

Eosinophils can initiate direct contact with cancer cells. Electron microscopy of TATE in carcinoma shows eosinophils in close proximity to tumor cells, indicating potential cross-talks between them [62]. Ultrastructural analysis of activated eosinophils depicts the EoSVs polarized toward cancer cells in support of their anti-tumorigenic role [63]. Activated eosinophils are shown to adopt NKG2D cytotoxic markers, which are more famously expressed on NK and CD8 + T cells, against cancer cells in some instances [63, 64]. Hollande et al. showed how post-translational modifications of chemokines regulate the trafficking of T cells and eosinophils to tumor sites and thus affect tumor growth [58]. They sought to elucidate the effect of a serine exopeptidase present on most cells called dipeptidyl peptidase 4 (DPP4) that truncates certain chemokines and is upregulated in cancer. The administration of sitagliptin, a DPP4 inhibitor (DPP4i), significantly reduced tumor volume. This DPP4i enhanced eosinophilia in solid tumors, and anti-Siglec-F-mediated eosinophil depletion significantly reversed sitagliptin’s tumor suppressive effect. Sitagliptin induced TATE by preventing DPP4-driven CCL11 truncation. The tumor lysates had elevated EPX and ECP concentrations, further proving a direct response to cancer cells. Further investigation showed that IL-33 is not only an inducer of CCL11 production and a protector against DPP4, but it also increases eosinophil-mediated tumor cytotoxicity. Although sitagliptin was still able to reduce tumor volume when anti-CD4/anti-CD8 was used to deplete T cells, the coexistence of T cells with eosinophils yielded the most optimal result, indicating a synergistic effect between T cells and eosinophils. Additionally, eosinophil depletion in T cell-deficient *Rag2*^*−/−*^ mice negated sitagliptin’s effect as well. The authors achieved the same anti-tumorigenic results after the injection of anti-Thy1 (CD90) antibody in *Rag2*^*−/−*^ mice to exclude the involvement of NK cells in T cell-deficient mice [58]. These results indicate the direct tumoricidal impact of eosinophils even in the absence of T or NK cells. The following sections will elaborate on how eosinophils can suppress or promote cancer cell growth through other immune cells and mediators.

### T cells

T cells constitute the foundation of cell-mediated immunity and are crucial for antigen-specific tumor elimination. T cells boast a range of subsets whose direct or indirect effect on tumor growth is suppressive (CTL, Th1), ambivalent (Th2, Th17), or supportive (Treg) [65]. The interactions of tumor-specific T cells with recruited eosinophils have recently been investigated, and they are found to be synergistic.

In an experimental model, Reichman et al. showed that activated eosinophils injected in eosinophil-deficient, ΔdblGATA mice suffering from colorectal cancer (*Apc*^*Min/+*^) were recruited to tumor sites and survived up to three months. Such tumor-mediated prolonged survival was reliant on tumor-secreted CCL11 but independent of IL-5 since anti-IL5-treated mice had eosinophils present in their colons [66]. Depletion of eosinophils in *Apc*^*Min/+*^ (*Apc*^*Min/+*^/ΔdblGATA) mice cut their survival in half compared to the *Apc*^*Min/+*^ control group, and the subsequent depletion of CD8 + T cells caused another 16% drop in *Apc*^*Min/+*^/ΔblGATA survival and increased tumor burden, which indicated a synergistic yet CD8 + T cell-independent anti-tumor effect from activated eosinophils. According to subsequent proteomics analyses, activated intra-tumoral eosinophils were abundant in proteins related to cell survival (BAX, BCL2, caspase-3) and Th1 cytokines and cell-surface receptors (IL-12, IFN-γ, CD44, CD79) compared to naïve eosinophils. IFN-γ-mediated signaling pathway was the most enriched in intra-tumoral eosinophils as opposed to naïve eosinophils, suggesting the role of this cytokine in potentiating eosinophils. These results were corroborated by a transcriptomic analysis that showed upregulation of genes such as *Stat1*, *Nos2*, and *Il12b* in infiltrated eosinophils. In vitro, IFN-γ was essential to increase the cytotoxic activity of eosinophils against colorectal tumor cells but not breast cancer cells [66]. The synergy of eosinophils and CD8 + T cells was previously shown in another study where eosinophils enhanced CD8 + T cells infiltration [67]. Infiltration of immune cells was dependent upon Treg depletion that resulted in TATE and enhanced leukocyte recruitment—especially CD8 + T cells and dendritic cells—as well as reducing B cell and macrophage infiltration. Anti-Singlec-F-mediated depletion of eosinophils significantly impeded tumor rejection and infiltration of CD45 + leukocytes, particularly CD8 + T cells. The above-mentioned Treg depletion induced the production of IFN-γ, TNF-α, nitric oxide synthase 2, and granzyme B as well as CCL5, CCL11, CXCL9, and CXCL10 chemokines in the TME. In contrast, eosinophil depletion drastically dropped the expression of these mediators, which indicates that activated eosinophils are tumoricidal and attract CD8 + T cells. The transfer of eosinophils alone, CD8 + T cells alone, or inactivated eosinophils suppressed tumor growth in mouse models but not significantly. Co-transfer of activated eosinophils and CD8 + T cells, where TATE occurred mostly in the tumor and also helped lodge tumor-specific effector T cells, led to substantial tumor growth inhibition and mouse survival time. Such ameliorating effects also caused vasculature normalization, diminished hypoxia and promoted normoxia, decreased vascular leakiness, and M1 macrophage polarization in the TME [67]. Vessel normalization alleviates tumor hypoxia and immunosuppression by inhibiting Treg accumulation and M2 macrophage polarization [68, 69]. The same positive results were reported in another breast cancer-driven lung metastasis experimental model where eosinophils were actively recruited to the metastatic TME independent of CCR3 and possessed varying degrees of degranulation [9]. Depletion of eosinophils in ΔdblGATA mice increased tumor burden, and the cellular composition of the lungs showed lower CD4 + and CD8 + T cell infiltration. Transcriptomic analyses revealed increased expression of activation-associated surface markers (*Cd86 Cd69*) and T/NK cell recruiting chemokines (*Ccl5, Cxcl9, Cxcl16*) plus an enrichment in IFN-γ, TNF-α, IL1-β signaling pathways in activated eosinophils compared to naïve ones. IFN-γ/TNF-α-activated eosinophils possessed superior tumoricidal effect and, while the source of TNF-α was unknown, CD4 + T cells, NK, and NKT were the main sources of IFN-γ. Overall, it seems the presence of IFN-γ-activated eosinophils augments and activates T cells in a type 1 immunity fashion.

### Plasma cells

PCs are B cell-derived specialized cells that produce antibodies. While mostly found in the bone marrow (BM), PCs are located in other organs as well. Eosinophils can mediate long-lived PC survival in the BM under baseline and post-immunization conditions [70]. The second most common hematologic malignancy, multiple myeloma (MM), is an incurable monoclonal gammopathy that is primarily characterized by excess PC production [71]. Apart from BM stromal cells (BMSCs), which support malignant PC proliferation, eosinophils are reported to enhance this process as well. A preliminary, in vitro study stated that eosinophil co-culture contributed to the proliferation of half of the tested PC cell lines and primary CD138 + MM cells [72]. The study also claimed that eosinophil-mediated PC support is independent of IL-6 and APRIL but failed to discern what factor increased PC proliferation. Wong et al. later revealed that IL-6 or APRIL supported MOPC315.BM myeloma cell growth in vitro but were redundant in high cell densities [73]. Eosinophils and megakaryocytes (a constitutive source of IL-6) co-localized with MOPC315.BM cells. Eosinophil depletion halted MOPC315.BM cell growth in BALB/c mice, but only in the early stages. The independence of myeloma cells from eosinophils in later stages could be due to cell-cell contact of myeloma cells in high cell densities or the autocrine production of IL-6 or APRIL cytokines by myeloma cells [74]. This would explain the discrepancy between the discussed experiments and why eosinophils are needed only in the early stages when myeloma cells are few. It should be noted that osteoclasts, macrophages, BMSC, etc. are also sources of IL-6, which makes the interpretation of eosinophils’ role in MM progression difficult [75].

### Natural killer cells

NK cells are an important component of the innate immune system and are currently being researched due to their rapid anti-tumor response, especially compared to T cells. Eosinophils enhance NK cell cytotoxicity. Korean Red Ginseng (KRG) is an immune-enhancing drug that augments the immunotherapeutic effect of NK-92 cells against metastatic liver cancer cells through eosinophils [76]. Of all leukocytes, this combination increased only eosinophil counts and subsequently MBP and IFN-γ production after increasing IL-33 levels. KRG activated NK cells and eosinophils through IL-33 which proceeded to secrete IFN-γ and MBP, respectively.

### Microbiota

Microbiota, the collective microorganisms in the body, and their alterations are implicated in inflammatory disorders, autoimmunity, and carcinogenesis. Organ- or TME-specific microbiota alter responses to cancer therapy and immunotherapy [77, 78]. Calcinotto et al. suggested a direct causative link between the presence of *Prevotella heparinolytica*—a gut microbe—and the local and distant differentiation of α_4_β_7_ integrin-expressing Th17 cells that can migrate to extra-mucosal sites and support malignancies. The study demonstrated elevated CD4 + T cell differentiation to IL-17-producing Th17 cells in the gut and then BM of Vk^*^MYC mice. Similar to IL-6, the over-expressed IL-17 heightened the incidence of MM occurrence by activating Vk^*^MYC PCs through STAT3 pathway [79]. Eosinophils accumulated in the BM as MM progressed and MCP3 (CCL7) levels grew. BM eosinophils expressed surface IL-17RA and IL-17RC and produced TNF-α and IL-6 upon IL-17 or MCP3 stimulation. IL-17 F is capable of inducing IL-1β and IL-6 proinflammatory cytokine production in eosinophils [80]. Interestingly, anti-IL-17RA and anti-IL-17 A injection only slightly decreased eosinophil recruitment which indicates the presence of another chemotactic factor [79]. And administration of anti-IL-5 alone did little to curb MM progression or Th17 recruitment to the BM, which suggests eosinophils’ pro-tumorigenic effect was at best mediatory and was indirect. Or, as was the case with other studies, eosinophil presence is unnecessary in late-stage MM development [72, 73].

### Mediators

*IL-5* As discussed, IL-5 is a major proponent of eosinophil proliferation and differentiation in the BM. It maintains B1 B cells and mucosal IgA production as well. As a Th2 cytokine, IL-5 is mainly secreted by CD4 + T and ILC2 cells and, to a lesser extent, by basophils and eosinophils [81]. Apart from its role in the pathogenesis of asthma, allergy, parasitic infection, and hypereosinophilic syndromes, the IL-5/eosinophil axis is reportedly correlated with both tumor suppression and progression. IL-5-producing cells expand when treated with IL-25 and more so with IL-33 under Th2-skewing conditions, and IL-5 neutralization impairs eosinophil recruitment and increases tumor lung metastasis. Reversing this effect with exogenous IL-5 treatment induces eosinophilia and suppresses metastasized melanoma cells’ expansion and proliferation [81]. In contrast, Zaynagetdinov et al. reported that IL-5 promotes lung metastasis but has no effect on the size of primary or metastatic tumors [82]. IL-5 itself had no direct effect on tumor proliferation but regulated the development of niches in metastatic sites that were conducive to tumor cell invasion through Tregs. The number of lung-infiltrated eosinophils in IL-5 knockout (KO) mice was much less than that of wild-type (WT) mice. Subsequently, the injection of BM-derived eosinophils into the bloodstream of IL-5 KO and WT mice increased the number of metastases in both groups. Further investigation into the underlying mechanism of this phenomenon showed a lack of CD8 + T cell recruitment in the lungs due to abundant eosinophil-secreted CCL22 that recruited FoxP3 + Tregs whose depletion drastically lowered metastasis. High CCL22 mRNA expression in WT mice eosinophils and unchanged TGF-β/IL-10 concentrations meant Tregs were recruited and not differentiated from native CD4 + T cells. The CCL22-driven Tregs also suppressed NK cells, CD4 + and CD8 + T cells’ IFN-γ production and reduced M1 macrophage polarization and MHC-II expression. Interestingly, IL-4 expression in the IL-5 KO mice was similar to that of WT mice, but IFN-γ levels were almost three times higher in the IL-5 KO mice, which showed how Th1 suppression favored tumor metastasis [82]. The massive drop in IFN-γ concentrations in the TME is suggestive of how the simultaneous dominance of type 2 immunity and abolishment of type 1 immunity would promote tumor progression. In such a pro-tumorigenic milieu, the activation of IL-5Rα downstream STAT5 protein and the down-regulation of NKG2D cytotoxic receptors are expected in eosinophils [82]. The reported spike in IL-33 concentration in the former study, which prevented excessive type 2 immunity skewing, could be why eosinophils demonstrated anti-tumorigenic function [81].

*IL-33* As an epithelial-derived cytokine, IL-33—a member of the IL-1 cytokine family—is implicated in allergy, autoimmunity, and inflammation. Under pathological conditions, IL-33 stimulates eosinophil precursor maturation and expansion in unison with IL-5 via its receptor ST2 and also promotes IL-5Rα expression on the precursor cells. IL-33 is secreted from damaged epithelial cells, activated immune cells, or even tumor cells. The mechanism of action of this alarmin is still debated [83]. While IL-33 promotes tumor progression in some cancer types, such as breast or colon cancer [84, 85], some studies have shown it to increase CD8 + T cell infiltrations and suppress melanoma cell growth or activate NK cells [86, 87]. Our review will focus only on the IL-33/eosinophil axis. IL-33 is responsible for the recruitment of eosinophils, T cells, and NK cells. It induces CCL11 expression, which results in TATE at tumor sites, and increases eosinophil anti-tumor function [85]. Lucarini and colleagues reported that IL-33-treated tumors become less populated with myeloid-derived suppressor cells (MDSCs) and myeloid DCs (mDCs) and, instead, IL-33 recruits CD8 + T cells and eosinophils [59]. Exogenously administered IL-33 increased the expression of alarmins, Th2 chemokines (CCL17 and CCL22), CD8 + T cell chemokines (CXCL10, CX3CL1), eosinophil chemokines (CCL11, CCL13, CCL24), as well as Th2-related cytokines (IL-4, IL-13) and Th1 effector molecules (granzyme B) in the primary tumor. The subsequent increased expression of CD107a and IFN-γ effector molecules in CD8 + T cells and NK cells plus balanced Th1 and Th2 cytokines revealed the antitumor role of IL-33. Eosinophil depletion significantly reduced the IL-33-mediated antitumor function and was parallel to reduced CD8 + T cell recruitment and lower CD8 + T cell and NK cell activation. This suggests that eosinophils indirectly inhibit primary tumor growth by T cells. In metastatic sites, eosinophil depletion also resulted in lower Th1-related genes and the formation of metastases without altering the immune cell composition of the metastatic sites. This establishes the role of eosinophils in IL-33-induced metastasis prevention and the maintenance of Th1 response. Significantly higher granzyme B, IFN-γ, and TNF-α production of IL-33-activated eosinophils in metastatic sites compared to unstimulated ones or eosinophils terminally differentiated by IL-5 was a testament to their direct tumoricidal activity and how IL-5 alone might not be sufficient for such a function [59]. Similar results were achieved in another study where IL-33 activated NK cells in the blood and recruited them to the TME through CCL5 to suppress metastatic tumor development [88]. CCL5 in the TME is partially produced by IL-33-activated T cells and eosinophils, which were also anti-tumorigenic. While depletion of eosinophils or CD8 + T cells alone had no effect on metastatic tumor development, simultaneous elimination of both cells significantly reversed IL-33-mediated tumor growth, further showing the cooperation of these cells in tumor suppression [88]. The source, location, and concentration of expressed IL-33 and IL-5 levels might explain the reason behind its different tumor-related effects. For example, IL-33 pre-treatment is shown to reduce CD8 + T cell cytotoxicity, increase PD-1, KLRG-1, and CTLA-4 inhibitory receptors, and recruit myeloid suppressor cells and FoxP3 + Tregs despite enhancing eosinophil and NK cell function (IL-5 not reported) [89]. Future studies could investigate how different levels of IL-5 and IL-33 contribute to eosinophil plasticity in cancer.

*GM-CSF* GM-CSF controls different aspects of eosinophil differentiation and biology along with IL-5 and IL-3 and triggers eosinophil anti-tumorigenic activities as well. Arnold et al. showed that GM-CSF, produced by CD4 + T cells and tumor cells, enhanced eosinophil anti-tumor responses [61]. Eosinophils readily infiltrated tumors, and their numbers were inversely associated with tumor volume. In the absence (PHIL mice) or excessive (IL-5-transgenic mice) presence of eosinophils, IFN-γ/TNF-α production of T cells was significantly lower and higher than in WT mice. This was because T cells were retained in the lymph nodes and failed to enter the TME in PHIL or IL-5-depleted mice, highlighting the role of eosinophils in T cell attraction and activation. The administration of recombinant GM-CSF and/or IL-5 in tumor-bearing mice significantly reduced tumor size, and no cytokine was more efficient than the other nor synergistic. The abrogation of GM-CSF signaling phenocopied the above-mentioned effects of IL-5 or eosinophil depletion, suggesting the anti-tumor effect of GM-CSF and how it resembles that of IL-5. This was because GM-CSF increased the expression of T-cell chemotactic cytokines such as CCL17 and CCL22. The phosphorylation of interferon regulatory factor 5 (IRF5) transcription factor is required for GM-CSF downstream signaling and thus eosinophil activation (Siglec-F/CD11b). IL-10 and its critical transcription factor, STAT3, interrupt the GM-CSF/IRF5 axis by reducing IRF5 phosphorylation. Almost 80% of the IL-10-expressing cells were monocytes and macrophages, and Foxp3 + Tregs constituted only 2% of the population. IL-10 suppresses both NK cells and T cell functions. IL-10R-deficient eosinophils were able to express excessive amounts of CCL5 [61]. GM-CSF from other sources can also stimulate anti-tumor functions in eosinophils. ILC2-derived GM-CSF recruits and enhances eosinophils to counter melanoma growth [90]. These cells express programmed cell death receptor (PD-1) inhibitory molecules similar to T cells. By activating ILC2 cells via IL-33, GM-CSF levels increased, resulting in eosinophil migration and activation. PD-1 expression, however, also increases in activated ILC2 cells. Thus, by inhibiting PD-1 in combination with IL-33, Jacquelot et al. found they could boost eosinophil and subsequently T cell activation to control melanoma tumor progression [90]. As evidenced by these results, GM-CSF imposes a negative effect on tumor growth and size, which is on par with that of IL-5, and also promotes eosinophils and T cells’ infiltration in the TME.

*TSLP* This cytokine is released from damaged normal and cancer cells, which tilts Th1/Th2 toward a type 2 immune response. Xie and colleagues investigated the correlation between TSLP and tumor growth in vitro [91]. Hypoxia promoted eosinophil recruitment and increased TSLP secretion from cervical cancer cells. Although eosinophil recruitment was attributed to TSLP, the presence of other chemotactic factors in the culture supernatant was neglected since the addition of anti-TSLP did little to lower eosinophil recruitment. Among different chemokines, cancer cells secreted CCL17 the most, whose concentration was directly correlated with eosinophil recruitment. Because anti-TSLP pre-treatment decreased CCL17 production, hypoxia was deemed to promote CCL17 production through TSLP. In vitro, treatment of eosinophils with TSLP decreased their CD80/C86 expression without altering HLA-DR levels. This treatment had no effect on type 1 (IFN-γ, TNF-α) cytokines, but significantly increased type 2 (IL-4, IL-5, IL-10, IL-13) cytokine concentrations. The co-culture of these polarized eosinophils with fresh cervical cancer cells increased their proliferation while inhibiting apoptosis [91]. This is evidence that hypoxia-induced TSLP stimulates eosinophil pro-tumorigenic functions by heavily biasing type 2 immunity in vitro, which could be applied to in vivo conditions as well.

*CCL11* CCL11/eotaxin-1 is crucial in eosinophil migration, TATE, and their subsequent tumoricidal activity [92]. Tumor-secreted CCL11 can recruit eosinophils to tumor cells and inhibit tumor growth. CCL11-overexpressing MS-K sarcoma cell clones (MS-K-CCL11) demonstrated higher eosinophil recruitment and BM eosinophil differentiation [92]. CCL11-induced eosinophils exhibited cytotoxic effects in co-culture with NFSA sarcoma cell lines and endothelial cells. CCR3-specific blockade in NFSA tumor-bearing mice increased tumor angiogenesis and blocked eosinophil infiltration, but no significant change in tumor weight was seen. The effect of eosinophil absence on restoring tumor blood vessel formation was indicative of how eosinophils would damage tumor vessels, or as previously discussed, normalize them.

## Cancer immunotherapy

Cancer immunotherapy, where the immune system components are manipulated to specifically target cancer cells, is a rapidly growing field of study. This modality has been successful enough to be deemed the fourth pillar of cancer care just behind surgery, chemotherapy, and radiotherapy. Based on the immune system component, immunotherapy is branched into different offshoots. The field has seen many recent breakthroughs, especially in the past decade, with the clinical approvals and promising results of adoptive cell therapy (ACT), immune checkpoint inhibitor antibodies (ICIs) and monoclonal antibodies, oncolytic virus therapy, cancer vaccines, and cytokine therapy [93]. ICIs and ACT are the most prominent members of this family whose relations with eosinophils has recently been investigated. The mechanistic role of eosinophils in cancer immunotherapy is only recently gaining attention. Eosinophil count is generally associated with immune-related adverse events (irAEs) [94] following ICB therapy but mostly a good prognosis (Additional file 1).

### Immune checkpoint blockade therapy

Immune checkpoints, such as cytotoxic T-lymphocyte antigen-4 (CTLA-4) and programmed cell death protein-1 (PD-1), are naturally occurring receptors on immune cells that regulate the immune response. PD-1 and CTLA-4 are present on immune cells, especially T cells, and bind to their respective ligands to suppress immune cells’ activation and expansion. This induces cell exhaustion to prevent overactivation of the immune cells and autoimmunity. Tumor cells hijack and exploit this pathway by expressing PD-1/CTLA-4 ligands to shut down cellular immunity and evade immune surveillance. Immune checkpoint blockade (ICB) therapy employs ICIs to target and block CTLA-4 and/or PD-1 receptors on immune cells or their ligands—CD80/CD86 and PD-L1—on tumor cells to enhance T cell activation and antigen-specific response [95]. Although ICB is an effective treatment modality, its response rates range from 10 to 50% in different solid tumors [96]. It also fails to yield positive results in non-immunogenic tumors such as triple-negative breast cancer. Since ICB boosts primarily T cell responses, and T cells and eosinophils have shown intimate cross-talk, the role of eosinophils in ICB therapy is under investigation.

**Fig. 3:**
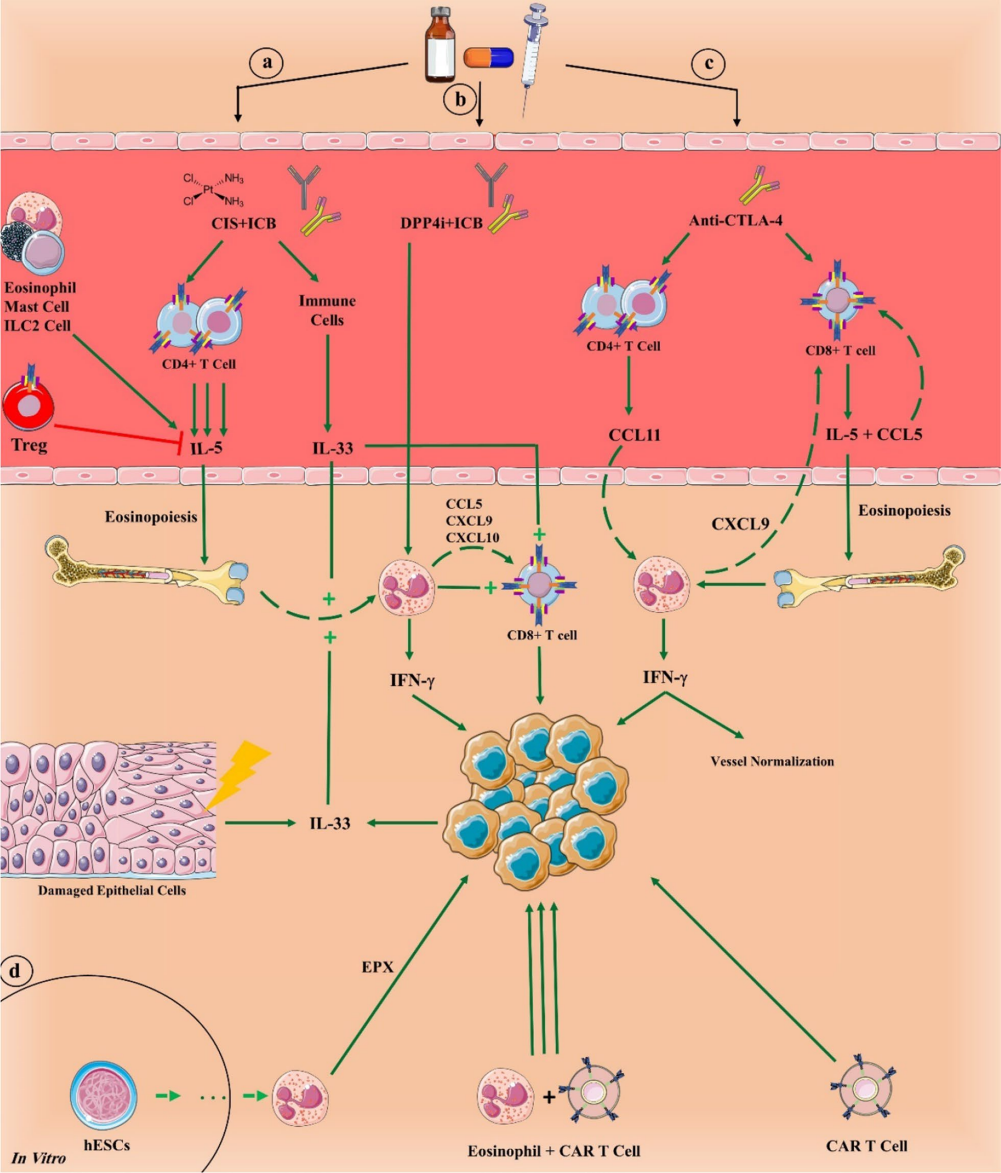
Eosinophils take part in a successful cancer immunotherapy. Treatment with immune checkpoint blockade (ICB) therapy and cisplatin (CIS) increases IL-5 and IL-33, which increase eosinophil differentiation and recruitment, respectively (**a**). Dipeptidyl peptidase-4 inhibitor (DPP4i), such as sitagliptin, actively stimulates eosinophils when combined with ICB (**b**). Anti-CTLA-4 alone is also capable of activating cellular immunity with CD4 + and CD8 + T cells and galvanizing eosinophils to eliminate tumor cells in synergy with T cells (**c**). In vitro differentiation of eosinophils from human embryonic stem cells (hESCs) could be vital strategy to create a product of eosinophils for immunotherapy. Both stem cell-differentiated eosinophils and chimeric antigen receptor (CAR) T cells can suppress tumor growth, but when combined, they synergize their tumor-killing potential (**d**). *ICB* anti-PD-1 plus anti-CTLA-4

A recent study sought to elucidate the role of eosinophils in ICB treatment in a non-immunogenic primary and metastatic breast cancer mouse model [97]. Only the triple administration of anti-PD-1, anti-CTLA-4, and cisplatin (ICB + CIS) engendered durable responses in mice, and eosinophils were the only cells that consistently increased after ICB + CIS therapy in the primary and metastatic tumor, the blood, and the healthy BM (Fig. 3). RNA-seq identified enrichment for IFN-γ pathway genes in infiltrated eosinophils. While eosinophil depletion had no effect on tumor growth in the cisplatin-alone group, it significantly negated the effect of ICB + CIS at primary and metastatic sites. This was attributed to the inactivation of intra-tumoral CD8 + T cells after eosinophil depletion. The plasma cytokine profiles of ICB + CIS- and ICB-treated mice showed IL-5, whose main source was traced back to CD4 + T cells and not ILC2 cells, to be the only cytokine significantly increased in the plasma. IFN-γ ranked as the second highest cytokine. IL-5 blockade had the same effect as anti-Siglec-8 treatment by dropping systemic and intra-tumoral eosinophil numbers. In ICB + CIS cases with the highest intra-tumoral eosinophil penetration, IL-33 and then TSLP were the most increased cytokines in the blood and tumor. IL-33 neutralization had no effect on systemic eosinophil count but hindered intra-tumoral eosinophil accumulation. This resulted in inhibited intra-tumoral CD8 + T cells activation and infiltration, and increased primary tumor growth [97]. Whether IL-33 directly recruits and/or activates T cells was undetermined, but its positive effect was previously demonstrated in immunogenic tumor models [98]. IL-33 production upregulates PD-1 and/or PD-L1 in tumor cells as well as T cells, NK cells, and ILC2 cells [89]. Although this would mean immune cells become more susceptible to tumor-mediated suppression, it would also open a window to block these receptors and enhance immune cells’ function. These studies show the importance of IL-33 and IL-5 co-expression in efficient eosinophil-T cell tumor suppression and how, at least in non-immunogenic tumors, eosinophils are necessary for T cell activation even in the presence of ICIs.

DPP4i, such as sitagliptin, could be an addition to ICI therapy. While PD-1/CTLA-4 blocking prevents tumor growth in immunogenic tumors, triple therapy with DPP4i shrinks tumor size significantly more by recruiting eosinophils, which further solidifies the idea of T cell-eosinophil combination therapy. Simultaneous PD-1/CTLA-4 inhibition and eosinophil depletion with or without DPP4i results in out-of-control tumor growth, which denotes the crucial role of eosinophils in controlling tumor volume even when T cells are activated [58]. GM-CSF is another potent molecule that reduces tumor growth as effectively as anti-PD-L1 or anti-CTLA-4 antibodies. The fact that GM-CSF shows no synergistic effect with these ICIs would point to the supportive role of eosinophils for T cells [61].

Eosinophils and mononuclear cells surround regressing tumors after anti-CTLA-4 treatment [99]. Anti-CTLA-4 administration alone is capable of increasing systemic eosinophil count and eosinophil infiltration into the TME by stimulating *IL5* and *CCL5* expression in activated CD8 + T cells and *CCL11* in CD4 + T lymphocytes [100]. The recruited eosinophils produce IFN-γ and CXCL9. CTLA-4 blockade also increases *IFNγ* and *Cxcl9* expression in immunogenic tumor cells, which are crucial in type 1 immunity and vessel normalization as well as T cell recruitment. This gene expression alteration is not observed in non-immunogenic tumors. Thus, anti-CTLA-4 indirectly normalizes tumor vessels by increasing pericyte coverage and vessel perfusion and decreasing vessel density in an eosinophil-dependent manner. The recruitment of eosinophils, CD4+, and CD8 + T cells depends on each other.

### CAR T cells

CAR T cell therapy is a relatively novel and effective immunotherapy for a range of hematopoietic malignancies and solid tumors. Patient T cells are isolated and inserted with a genetically engineered *CAR* transgene to express CARs that are directed against a certain tumor antigen and then reinfused back into the patient [101]. CAR constructs guide T cells, or any other cell they are mounted on, to target a specific antigen. Based on CAR generation, different costimulatory domains such as 4-1BB (CD137), ICOS, and OX-40, are designed to provide co-stimulatory signals to activate CAR T cells [102]. Considering the synergistic effect of T cells and eosinophils, they could be an ideal cell to support CAR T cell responses against tumor cells, but the immunotherapeutic potential of eosinophil incorporation is scarcely investigated.

Cell or gene therapy can be greatly enhanced by reprogramming stem cells to produce a variety of cell types [103]. Lai et al. produced large quantities of functional human eosinophils from human embryonic stem cells (hESCs) and human induced pluripotent stem cells (iPSCs), which are comparatively easy to produce. Their protocol expanded eosinophils roughly 10,000-fold with more than 94% purity. In vitro, hESC-derived eosinophils exhibited highly specific anti-tumorigenic activities with no cytotoxicity to normal healthy cells (Fig. 3). In immunodeficient NPG mice preconditioned with HCT116 tumor cells, intravenously injected CD45 + hESC-derived eosinophils infiltrated the tumor, restricted tumor growth, and prolonged the median survival time of the mice in both inoculated tumors and established tumors. Interestingly, almost the same results were achieved after administering CAR-T cells alone. The simultaneous injection of CAR T cells and hESCs-derived eosinophils, however, showed a more significant reduction in tumor volume and extension of survival time compared with CAR T cells alone [104]. The positive role of eosinophils in CAR T cell function and efficient recruitment in a CXCL9/CXCL10-dependent manner was previously noted as well [105]. Such results corroborate other findings that posit activated eosinophils function in synergy with T cells to abolish tumor cells.

## Conclusions and future perspectives

Multiple studies, including the more recent research, show eosinophils to be a key component of orchestrating immune cells against tumors. In this review, we comprehensively categorized eosinophil-related factors in TME and emphasized the importance of designing a study that encompasses all the relevant cells (T cells, NK cells), mediators (IL-5, IL-33, CCL11), cytokines (IFN-γ, TNF-α) and transcription factors and signaling proteins (IRF, STAT), in order to achieve results that best reveal the role of eosinophils in cancer treatment. Cumulative studies of the past decade generally point to the anti-tumorigenic effect of eosinophils rather than a pro-tumorigenic role, both greatly determined by various signals in the TME. Similarly, neutrophil anti-tumorigenic activity as innate immune cells is dictated by different environmental cues of the primary or metastatic TME [106]. It seems that a Th1 or a balanced Th1/Th2 immune response is associated with eosinophil-mediated tumor suppression while an over-stimulated type 2 response that quashes type 1 immunity mediates tumor progression. The STAT signaling pathway in eosinophils correlates with what part they play in tumor fate. While STAT1 phosphorylation hinders tumor growth, STAT3 and STAT5 are involved in tumorigenesis [61, 66, 82].

Much of the discrepancy in the conflicting results of eosinophil pro- or anti-tumorigenic functions is possibly due to simple in vitro studies, different or inappropriate cancer models that artificially skew toward type 1 or 2 immunity, different tumors, analyzing primary or metastatic tumors, or the misrepresentation of TME settings. Some studies use chemically induced tumor cell lines that actively recruit eosinophils via enhanced levels of IL-5, CCL11, etc. [64, 107], while others examine the function of a single eosinophil mediator such as IL-33 or TSLP [59, 91] on simple cancer cell lines. Isolated in vitro, co-culture of eosinophils and tumor cells hardly depicts the complexity of TME [72]. Of course, establishing models with a dominant type 2 immune response will likely paint eosinophils as pro-tumorigenic cells [108]. Most of the earlier studies focused on eosinophils alone and not on eosinophils with other immune cells, such as T cells. The effect of eosinophils on tumors resembles a puzzle with different pieces that work together; the absence or exaggerated presence of a piece could skew the results. In the TME, innate immunity cells regulate adaptive immunity cells like T cells [109].

The number of infiltrating eosinophils is speculated to be another reason for a different tumor outcomes [9]. Eosinophils infiltrate various tumors in distinct numbers. For instance, the abundance of eosinophils in the colon and esophagus is high while fewer eosinophils are recruited to the lungs, ovaries, and breasts [110]. This also reflects in the tumor stage. Apparently, in the early stages of the tumor where eosinophils are few, they are anti-tumorigenic, aid in immune surveillance, and contribute to the Th1 response. As eosinophils become more numerous in the later stages, their excessive Th2 skewing shuts down the Th1 response, destabilizes the Th1/Th2 balance, and favors tumor progression as a result. Th1/Th2 equilibrium is key [81].

Another key point that could explain the dichotomy between the pro- or anti-tumorigenic activity of eosinophils is the nature and uniqueness of a tumor. Eosinophils are consistently reported to exacerbate MM progression. Myeloma cells are central to plasma cell dyscrasia and thrive on IL-6 and APRIL. Though eosinophils secrete these cytokines, other BM cells eclipse eosinophil IL-6 production, making eosinophils more or less redundant. BMSCs that support myeloma cell growth, macrophages, and osteoclasts are prominent IL-6 producers [73, 111]. Cell-to-cell contact, which escalates as myeloma cells expand, is another determinant of MM outcome [71]. Thus, it is unsurprising that eosinophils are pro-tumorigenic in these types of malignancies. The very environment in which eosinophils are recruited is equally important. The condition of TME, its produced cytokines, and eosinophil exposure to innate or adaptive immune cells alter the polarization of eosinophils, leading to tumor progression or retardation. Other cells of the innate immune system, such as neutrophils and macrophages, similarly have diverse roles that are dictated by their environment [112, 113]. The mere existence of inactive eosinophils is inconsequential to tumor suppression, and type 1 polarization and tumor-specific T cell infiltration is required. Eosinophil activation status seems to be of importance as well. Unlike earlier studies that overlooked this state, most of the recent studies that concluded these cells were anti-tumorigenic used omics analyses to discern eosinophil activation via IFN-γ/TNF-α. While type 2 immunity can also hinder tumor growth, an overactive Th2 status that minimizes Th1 activities tends to support tumor progression. The supportive influence of eosinophils on helper or cytotoxic T cells, which are arguably the most potent anti-tumor cells, has been shown on multiple occasions [9, 58, 59, 61, 66, 67, 97, 100, 104]. It seems that, when activated, eosinophils can reshape TME structure and exert their supporting role for other immune cells, particularly CD8 + CTLs [67].

Most controversial studies on this topic are related to eosinophil-related cytokines. Multiple cytokines in the TME work in concert to determine clinical outcomes. Assessing IL-5 alone in type 2 immunity settings, for instance, and not evaluating other tumor-related mediators, such as IL-33, would paint eosinophils as cancer promoters [108]. The concentrations of cytokines should be taken into account as well. Introducing high or low levels of exogenous cytokines or transplanting mice with tumors that overexpress certain cytokine genes will only engender misleading results. The same holds true for TME cells; simply evaluating eosinophil interactions with one cell type will lead to the misinterpretation of the results.

Eosinophils are showing promising results in immunotherapy settings, especially in ICB and CAR T cell therapies [58, 61, 97, 100, 104]. This comes as no surprise since eosinophils support T cells’ recruitment and activation. Their synergy with CAR T cells and ICIs, lack of an endogenous T cell receptor (TCR) that bypasses graft-versus-host disease, and their rapid response by virtue of being innate immune cells that quickly infiltrate tumors and attract other immune cells make them a potential adjuvant therapy. Their number limitations can be overcome by utilizing HSCs as a source, optimizing in-lab expansion, or using suitable replacement cell lines. Such features have made allogeneic NK cells, CAR-NK cells, and CAR-macrophage (CAR-M) cells an emerging trend in adoptive cell therapy. CAR-NK and CAR-M cells are being researched as a substitute or complement therapy to CAR-T cells [114]. Perhaps CAR-eosinophils can be investigated as a complement to CAR-T cell therapies.

The paucity of data is another reason for conflicting data on this issue. Bulk cell analysis has only recently been used to investigate eosinophils in cancer therapy. According to our research, no study has applied single-cell analysis in this context. Single-cell analysis is a powerful burgeoning omics tool that can be used at different molecular levels (e.g. genomics, transcriptomics, proteomics, etc.) to find valuable data regarding various tumors or the cell product, such as CAR-T cells. Single-cell RNA sequencing, for instance, is able to classify the cells of a tumor or the therapeutic cell product into distinct clusters based on their transcriptome similarity. It can also reveal what gene groups are up- or down-regulated. Such a tool can be utilized to distinguish eosinophils before and after interacting with different tumors to discern which eosinophil groups—based on their genetic expression—were anti- or pro-tumorigenic and which genes were involved. Perhaps, such genes can be the target of the CRISPR/Cas9 gene editing platform that is used to boost adoptive cell therapy now more than ever [115]. Single-cell analysis can also identify eosinophil subtypes beyond the conventional flow cytometric analyses. Future studies should also track the changes and interactions of innate and adaptive immune cells with eosinophils and examine their potential impact on the pro- or anti-tumorigenic function of eosinophils. A deeper knowledge of eosinophils’ inner workings could turn eosinophils into a potential immunotherapeutic agent either alone or as a combination therapy. Future studies can focus on turning eosinophils into a weapon against tumor cells parallel to further studying their part in TME and cancer outcomes.

### Supplementary information

**Additional file 1.** The prognostic value of eosinophil-related indixes in cancer and cancer immunotherapy.

## Data Availability

Not applicable.
